# Changes in affect after completing a mailed survey about trauma: two pre- and post-test studies in former disability applicants for posttraumatic stress disorder

**DOI:** 10.1186/s12874-017-0357-x

**Published:** 2017-05-10

**Authors:** Maureen Murdoch, Shannon Marie Kehle-Forbes, Melissa Ruth Partin

**Affiliations:** 1 Section of General Internal Medicine, Minneapolis VA Health Care System, Minneapolis, MN USA; 2 Center for Chronic Disease Outcomes Research, Minneapolis VA Health Care System, Minneapolis, MN USA; 3 Department of Internal Medicine, University of Minnesota Medical School, Minneapolis, MN USA; 4 National Center for PTSD Women’s Health Sciences Division at VA Boston Healthcare System, Boston, MA USA; 5 Center for Chronic Disease Outcomes Research (111–0), Minneapolis VA Medical Center, One Veterans Drive, Minneapolis, MN 55417 USA

**Keywords:** Pre-post test observation, Patient surveys, Posttraumatic stress disorder, Ethics, Trauma, Research participation, Iatrogenic, Psychological distress, Affect, Research subjects/psychology

## Abstract

**Background:**

One potential concern with using mailed surveys containing trauma-related content is the possibility of re-traumatizing survivors without a trained mental health professional present. Prior research provides insufficient guidance regarding the prevalence and magnitude of this risk because the psychological harms of trauma-related surveys have typically been estimated using single post-test observations. Post-test observations cannot quantify magnitude of change in participants’ emotional states and may over or under estimate associations between participants’ characteristics (risk factors) and post-survey upset.

**Methods:**

We conducted two pre- and post-test studies in samples of former applicants for posttraumatic stress disorder disability benefits: 191 males who served during Gulf War I plus 639 male and 921 female Veterans who served sometime between 1955 and 1998. We used two 9-point items from the *Self-Assessment Manikins* to measure participants’ valence (sadness/happiness) and arousal (tenseness/calmness) before and after they completed mailed surveys asking about trauma-related symptoms or experiences. We examined the following potential predictors for post-survey sadness and tenseness: screening positive for posttraumatic stress disorder, having a serious mental illness, and history of military sexual assault or combat.

**Results:**

After the survey, across the groups, 29.3–41.8% were sadder, 45.3–52.2% had no change in valence, and 12.9–22.5% were happier; 31.7–40.2% were tenser, 40.6–48.2% had no change in arousal, and 17.3–24.0% were calmer. The mean increase in sadness or tenseness post-survey was less than one point in all groups (SD’s < 1.7). Cohen’s *d* ranged from 0.07 to 0.30. Most hypothesized predictors were associated with greater baseline sadness or tenseness, but not necessarily with larger post-survey changes. Women with a history of military sexual assault had the largest net post-survey changes in sadness (mean = 0.7, SD = 1.4) and tenseness (mean = 0.6, SD = 1.6).

**Conclusion:**

While a substantial minority of Veterans reported more sadness or tenseness post-survey, the net change in affect was small. Most hypothesized risk factors were actually associated with higher baseline sadness or tenseness scores. When receiving unsolicited, trauma-related surveys by mail, separate protections for Veterans with the risk factors studied here do not seem necessary.

**Electronic supplementary material:**

The online version of this article (doi:10.1186/s12874-017-0357-x) contains supplementary material, which is available to authorized users.

## Background

Mailed surveys are among the most inexpensive methods available for collecting data from large, representative numbers of people. Moreover, when the research topic is sensitive, mailed or other self-administered surveys may generate more complete and accurate information compared to other data collection modes, such as face-to-face interviews [1–4]. In the United States, human studies involving mailed surveys may be approved under expedited Institutional Review Board (IRB) procedures if they meet “minimal risk” criteria [5]. According to the United States Code of Federal Regulations [6], “minimal risk” pertains if the research’s probability and magnitude of harm or discomfort does not exceed the risks of day-to-day living or the risks associated with physical or psychological examinations or tests (e.g., as might be encountered during a physician visit). However, expedited or no, many IRBs are reluctant to approve mailed surveys with sensitive content. Particularly when mailed surveys include items about prior traumatic experiences, some IRBs worry that selected survivors might be re-traumatized and clinically decompensate. Given the self-administered nature of mailed surveys, researchers cannot recognize when this has happened and hence cannot offer immediate interventions.

We know of only one randomized controlled trial to examine the risk of clinical decompensation after receiving a mailed survey with trauma-related content. In that study, Veterans with posttraumatic stress disorder (PTSD) randomized to receive the survey had reduced—not increased—mental health care visits in the 8 weeks post- survey compared to the 8 weeks pre-survey. This reduction was similar to that seen in the unsurveyed controls [7].

More typically, the probability of harm or discomfort from trauma-related mailed surveys has focused on survey participants’ emotional reactions using single post-test evaluations. For example, Newman et al.’s [8] *Reactions to Research Participation Questionnaire* (RRPQ), includes the following “upset” question: “Completing this survey upset me more than I expected.” Response options range from “Strongly Disagree” to “Strongly Agree.” In a systematic review in which the RRPQ’s use predominated, approximately 1 to 19% of research participants reported at least some immediate upset to survey questions about psychiatric symptoms, and about 1 to 32%, to questions about trauma [9]. While only a minority of participants reported upset or distress, the range was wide.

Despite providing important insight into survey recipients’ reactions, the RRPQ does not assess the degree of upset originally anticipated by participants, how upset they were before or after completing the survey, and, by extension, the total degree of upset induced by the survey. In other words, the RRPQ may speak to the probability of harm, but it does not speak to its magnitude. If up to a third of trauma survivors were to become unexpectedly upset when participating in trauma-related surveys, then understanding the magnitude of that upset becomes critical to weighing the harms and benefits of such research.

In the present study we used a pre- and post- test design in 2 randomly selected samples of trauma survivors to: 1) measure participants’ probability of experiencing more negative affect after completing a trauma-related survey compared to just prior to the survey, and 2) measure the magnitude of affective change, if any.

We were also interested to see if certain subgroups might be at particular risk of experiencing negative affect post-survey. Prior research relying mostly on single post-test evaluations suggests that individuals with PTSD diagnosis carry greater odds of post-survey upset to trauma-related surveys than other individuals [10]. We speculated that participants with serious mental illness might also be at increased risk of emotional upset. Lastly, we examined whether the type of trauma participants experienced –namely, combat or military sexual assault, affected their probability of experiencing post-survey upset. Although sexual assault history has been associated with greater odds of emotional upset post-survey in several studies using single post-test evaluations [7, 11–13] the association did not reach statistical significance in a recent meta-analysis [10]. Despite combat’s saliency to military Veteran populations, associations between combat exposure and post-survey upset have only rarely been addressed [7]. If, when using stronger study designs, subgroups with selected mental health disorders or trauma exposures demonstrate especially high risks or magnitudes of post-survey distress, then special, separate protections might be needed when surveying them.

## Methods

### Study design

We conducted a pre- and a post-test study in two independent samples. The Minneapolis VA Health Care System’s Internal Review Board (IRB) for Human Studies reviewed and approved both studies’ protocols. All analyses were pre-planned.

### Populations and setting

#### Sample 1

This study was undertaken at our local IRB’s request. In anticipation of a larger study, we randomly selected 324 men from the population of 46,824 male Gulf War I Veterans who applied for VA PTSD disability benefits prior to June 2007 and had served in the United States Armed Forces between August 2, 1990 and July 31, 1991. Data were collected between July 1, 2007 and July 31, 2008 and included several potentially sensitive questions pertaining to participants’ trauma exposures and subsequent mental health symptoms. Sample 1 will be referred to as the GWESt (*G* *ulf* *W* *ar 1* *E* *ra Veterans* *St* *udy*) sample or GWESt men. Overall, 196 (60.4%) of GWESt men responded to this survey.

#### Sample 2

The sampling frame was a representative, gender-stratified cohort of 871 men and 1,229 women who served in the US Armed Forces sometime between 1955 and 1998 and applied for VA PTSD disability benefits between January 1994 and December 1998. These cohort members were surveyed by mail about their PTSD symptoms and functioning between January 1, 2011 and December 30, 2012 (*Time 3*). They had previously completed surveys between 1998 and 2000 (*Time 1*) and 2004–2006 (*Time 2*) that asked extensive questions about their military and post-military trauma exposures and about their symptoms of depression and PTSD [14–17]. Approximately one tenth of the men and one fifth of the women in this sample had a serious psychiatric disorder [18]. Sample 2 will be referred to as the IMPROVe (*I* *nterviews to* *M* *easure* *P* *TSD* *R* *ecovery of* *Ve* *terans Study*) sample or IMPROVe men and women. 713 (81.9%) IMPROVe men and 1,015 (82.6%) IMPROVe women completed the Time 3 survey*.*

### Protocol

We mailed a study packet to GWESt men’s homes that included a cover letter describing the study’s risks and benefits, a $10 or $20 cash incentive, and 25-page questionnaire. At 2 week intervals, non-respondents were mailed a post-card reminder, second mailing of the questionnaire, and final mailing of the questionnaire via Federal Express overnight mail [19]. IMPROVe men and women received pre-notification letters to their homes followed by a study packet 1 week later that included a cover letter describing the study’s risks and benefits, a $20 cash incentive, and 20-page questionnaire. At 2 week intervals, non-respondents were mailed a post-card reminder, second mailing of the survey, and final mailing of the survey via United States Postal Service Priority Mail. In both studies, return of the survey signified consent to participate.

In both studies, the cover letters explicitly described the surveys’ content, warned participants that they might find some questions upsetting, and explicitly encouraged them to skip any upsetting questions or to opt out of the study completely if they were worried it might be too upsetting. We also provided Veterans with several copies of help-line numbers in their mailing packets, printed the same help-line numbers throughout the questionnaires’ pages, and posted them on the back page of the questionnaire. The questionnaires’ covers included a warning that some questions might feel too personal to the Veteran and informed them that they could skip any questions they didn’t wish to answer.

In both studies, the questionnaire was bound into a booklet so that all test items were presented in fixed order and could not be shuffled. Pre-testing of the two surveys suggested that most Veterans could complete a questionnaire within 30 to 45 min, though some Veterans required as much as 90 min.

### Measures

Although we used the same survey measures in both samples, in the IMPROVe study we assessed military sexual assault and combat exposures at Time 1 only and did not repeat those measures at Time 3. Chart diagnoses of serious mental illness were available for the IMPROVe sample only and were abstracted at Time 2.

### Main outcome: affect

According to the circumplex model of affective states [20], all emotions can be mapped along two orthogonal axes. One axis, “valence,” is anchored by happiness on one end and sadness or unhappiness on the other. The other axis, “arousal,” is anchored by tenseness (aroused) on one end and calmness (unaroused) on the other. We used the *Self-Assessment Manikins* (SAM) [21] to assess each participant’s sadness/happiness and tenseness/calmness immediately before and after completing the survey. For valence, a continuum of 5 stylized human figures (manikins) depicts feeling “very happy” (score of 1) to “very sad” (score of 9). For arousal, a second continuum of 5 stylized manikins depicts feeling “very calm” (score of 1) to “very tense” (score of 9). Although there are 5 manikins, intermediate response options allow participants to choose a feeling halfway between two manikins. Thus, there are a total of nine response options for both scales. In both studies, the survey’s first full page presented the valence and arousal manikins and asked participants to select “how happy or sad (or tense or calm) are you *right now*?” The two sets of manikins were repeated on the last full page of the survey, and participants were again asked to mark how happy or sad (or tense or calm) they felt “*right now*.” A one-unit change on either SAM item indicates a change halfway between manikins, whereas a two-unit change reflects movement from one full manikin to the next.

Veterans’ change in affect was calculated by subtracting post-survey SAMs from their pre-survey scores. Positive score changes indicate more sadness or tenseness, and negative scores, more happiness or calmness. Greater sadness or tenseness post-survey was considered undesirable. We created three affective score change categories to assess the possible post-survey outcomes: score changes > 0 = sadder or tenser –that is, undesirable change; 0 = unchanged; and < 0 = happier or calmer.

### Predictors or correlates of change in affect

#### Mental health disorders

In both samples PTSD symptoms were measured contemporaneously using the *Penn Inventory for PTSD* [22] and then dichotomized into positive versus negative screens using its published cut point. For IMPROVe only, we also had VA chart diagnoses of persistent serious mental illness between 1994 and 2006. Veterans were categorized as having persistent serious mental illness if they were diagnosed with bipolar disorder, schizophrenia, or schizoaffective disorder at least once in 3 separate calendar years.

#### Trauma exposures

We used a modified *Combat Exposure Index* [23] to assess combat exposure in both samples. The modifications added questions about combat experiences that were especially relevant to Gulf War I. Military sexual assault was assessed using three items from the military version of the criminal sexual misconduct subscale of the Sexual Harassment Inventory [24] plus a fourth Sexual Harassment Inventory item that assesses non-work related sexual assault*.* Responses were dichotomized into “any” versus “none.”

### Power

Sample sizes were fixed in both studies and no formal power analysis was done.

### Analysis

We limited analyses to the 191 GWESt men, 639 IMPROVe men, and 921 IMPROVe women who completed pre- and post-survey SAMs. IMPROVe men and women’s results are reported separately to account for IMPROVe’s stratified sampling strategy.

To examine the probability of experiencing more undesirable affect after completing a trauma-related survey, we report the percentage of participants within each sample who reported increased sadness or tenseness after completing their survey, as well as those who reported increased happiness or calmness or no change. Within each sample, results are reported overall and by each hypothesized correlate or predictor. We used *χ* ^2^ tests to examine the association between affective change category and the hypothesized correlates or predictors. Mean score changes and standard deviations (SD) describe respondents’ magnitude of change in sadness and tenseness overall and by each hypothesized correlate or predictor. We also calculated the overall Cohen’s *d* [25] for each sample. Finally, because changes in valence and arousal do not necessarily move in lock step, we report the cross-tabulation of participants’ change in valence versus their change in arousal. Pearson’s *r* describes the concordance of change between the two. We used SPSS version 19 for computations and a conventional *p* < 0.05 to denote statistical significance.

## Results

### Sample descriptives

As Table 1 shows, although both studies were comprised of former applicants for VA PTSD disability benefits, each sample was unique. Each group differed in the percentage with prior combat and military sexual assault experiences, for example.

**Table 1:** Characteristics of the two study samples, reported as a percentage (%) of the sample

Characteristic	Sample		
	GWESt	IMPROVe	
	Men	Men	Women
	*N* = 191	*N* = 639	*N* = 921
Sociodemographics			
Served during Vietnam Conflict	NA	89.3	24.1
Served after Vietnam but before Gulf War I	NA	4.2	35.3
Served during Gulf War I	100	5.8	30.6
Served after Gulf War I	NA	0.6	10.0
Age ≥ 50 years	44.3	92.5	62.5
White race	52.1	70.4	74.0
College education	74.5	59.2	88.1
Currently employed	50.8	7.8	27.0
PTSD screen positive	64.6	70.5	61.3
Military exposures			
Sexual assault	4.7	5.3	73.6
Combat	53.1	93.0	28.0

*GWESt* Gulf War I Era Veterans study, *IMPROVe* Interviews to Measure PTSD Recovery of Veterans study, *NA* not assessed, *PTSD* posttraumatic stress disorder

### Probability of undesirable affective change

As can be seen from Table 2, column 2, between 29.3 and 41.8% of the samples overall reported increased sadness post-survey, and 31.7 to 40.2% reported increased tenseness. 40.6 to 48.2% reported “no change” in affect, and 12.9 to 24% reported more happiness or calmness post-survey.

**Table 2:** Affective change category overall and by hypothesized correlate or predictor

Affective Change Category	Overall	By Hypothesized Correlate or Predictor							
		PTSD Screen Positive		Persistent SMI		Combat Exposure		Military Sexual Assault	
		Yes	No	Yes	No	Yes	No	Yes	No
Valence (Sadness/Happiness)									
GWESt Men									
Sadder	29.3	**32.7**	15.8*	NA	NA	**39.6**	18.0**	22.2	29.7
No Change	48.2	**43.8**	**65.8**	NA	NA	**42.6**	**53.9**	44.4	48.4
Happier	22.5	**23.5**	**18.4**	NA	NA	**17.8**	**28.1**	33.3	22.0
IMPROVe Men									
Sadder	31.1	33.6	24.9	29.8	31.2	31.3	26.7	29.4	31.1
No Change	52.2	50.1	57.1	54.4	51.9	51.6	60.0	50.0	52.3
Happier	16.8	16.3	18.0	15.8	17.0	17.1	13.3	20.6	16.6
IMPROVe Women									
Sadder	41.8	**46.9**	33.7***	40.2	42.2	41.5	41.9	**44.8**	33.3**
No Change	45.3	**40.4**	**53.1**	43.1	46.0	43.0	46.2	**43.5**	**50.2**
Happier	12.9	**12.7**	**13.2**	16.7	11.8	15.5	11.9	**11.7**	**16.5**
Arousal (Tenseness/Calmness)									
GWESt Men									
Tenser	35.4	**39.6**	18.4*	NA	NA	40.2	30.3	22.2	36.1
No change	40.6	**35.7**	**60.5**	NA	NA	37.3	44.9	33.3	41.0
Calmer	24.0	**24.7**	**21.1**	NA	NA	22.5	24.7	44.4	23.0
IMPROVe Men									
Tenser	31.7	**34.7**	24.3*	31.6	31.7	31.7	31.1	26.5	32.0
No change	48.2	**46.5**	**52.4**	49.1	48.0	48.2	48.9	52.9	47.9
Calmer	20.1	**18.8**	**23.3**	19.3	20.2	20.1	20.0	20.6	20.1
IMPROVe Women									
Tenser	40.2	**43.5**	34.8*	38.7	40.5	36.4	41.6	**44.2**	28.8***
No change	42.6	**39.6**	**47.2**	42.6	42.6	44.6	41.8	**40.9**	**47.3**
Calmer	17.3	**16.8**	**18.0**	18.6	16.9	19.0	16.6	**14.9**	**23.9**

*PTSD* posttraumatic stress disorder, *SMI* serious mental illness, *GWESt* Gulf War I Era Veterans study, *IMPROVe* Interviews to Measure PTSD Recovery of Veterans study, *NA* not available. **Bold face font** signifies statistically significant association between change in affective state post-survey and the hypothesized correlate or predictor. *p < 0.05, **p < 0.01, ***p < 0.001

Results Reported as Percentages (%)

A positive PTSD screen was associated with statistically significantly greater probability of being sadder post-survey in both GWESt men and IMPROVe women but not in IMPROVe men. All groups were more likely to report being tenser post-survey if they had a positive PTSD screen. Persistent serious mental illness was not associated with higher risk of post-survey sadness or tenseness in any group. Combat history was associated with a statistically significantly greater probability of being sadder post-survey in the GWESt men, as was military sexual assault history in the IMPROVe women.

### Magnitude of change

As Table 3 shows, across all samples, the mean increase in sadness or tenseness post-survey was less than one point or one “half-manikin” overall. The Additional file 1: Figure S1 shows the mean change in valence and arousal within each affective change category.

**Table 3:** Magnitude of affective change post-survey overall and by hypothesized correlates or predictors

Affect	Overall	Hypothesized Correlates or Predictors							
		PTSD Screen Positive		Persistent SMI by Chart		Combat Exposure History		Military Sexual Assault History	
		Yes	No	Yes	No	Yes	No	Yes	No
Valence (Sadness/Happiness)									
GWESt Men	0.1 (1.4)	0.3 (1.4)	−0.1 (1.4)	NA	NA	0.4 (1.4)	−0.2 (1.4)	−0.6 (1.6)	0.2 (1.4)
IMPROVe Men	0.3 (1.3)	0.3 (1.2)	0.1 (1.3)	0.3 (1.5)	0.3 (1.3)	0.3 (1.3)	0.2 (1.3)	0.2 (1.0)	0.3 (1.3)
IMPROVe women	0.6 (1.4)	**0.6 (1.4)**	0.3 (1.3)***	0.5 (1.5)	0.6 (1.3)	0.5 (1.5)	0.6 (1.3)	**0.7 (1.4)**	0.3 (1.4)***
Arousal (Tenseness/Calmness)									
GWESt Men	0.3 (1.7)	0.5 (1.7)	0.0 (1.5)	NA	NA	0.5 (1.7)	0.4 (1.9)	−0.6 (2.0)	0.4 (1.6)
IMPROVe Men	0.3 (1.4)	**0.4 (1.4)**	0.1 (1.5)*	0.3 (1.5)	0.3 (1.4)	0.3 (1.4)	0.4 (1.3)	0.2 (1.4)	0.3 (1.4)
IMPROVe women	0.5 (1.6)	0.5 (1.7)	0.4 (1.4)	0.4 (1.9)	0.5 (1.5)	**0.2 (1.6)**	0.5 (1.6)*	**0.6 (1.6)**	0.2 (1.5)***

*PTSD* posttraumatic stress disorder, *SMI* serious mental illness, *GWESt* Gulf War I Era Veterans study, *IMPROVe* Interviews to Measure PTSD Recovery of Veterans study, *NA* not available, **Bold face font** signifies statistically significant difference between those with and without the hypothesized correlate or predictor. *p < 0.05, ***p < 0.001

Results reported as Mean Score Change and Standard Deviation (SD)

The Additional file 2: Table S1 shows that many of the hypothesized predictors or correlates of undesirable affective change were actually associated with greater sadness or tenseness at baseline. After the survey, most individuals with these characteristics still had greater sadness or tenseness compared to their counterparts, but, as Table 3 shows, their mean score changes were less than one point and—for the most part—not significantly different from their counterparts’ mean score changes. Post-survey, IMPROVe women with positive PTSD screens or a history of military sexual assault had the largest net increases in sadness or tenseness, but the net change was again less than one point. Cohen’s *d* for change in valence was 0.07 for GWESt men, 0.14 for IMPROVe men, and 0.30 for IMPROVe women. Cohen’s *d* for change in arousal was 0.15 for GWESt men, 0.14 for IMPROVe men, and 0.23 for IMPROVe women.

### Concordance between changes in valence and arousal post-survey

As Table 4 shows, individuals’ changes in valence (sadness/happiness) post-survey did not necessarily move in the same direction as their changes in arousal (tenseness/calmness) in any of the samples. Pearson’s *r* between the changes in valence and arousal post-survey was 0.46 for GWESt men, 0.32 for IMPROVe men, and 0.44 for IMPROVe women (all *p*s < 0.001).

**Table 4:** Cross-Tabulation of Veterans’ Affective Changes Post-survey

	Sample											
	GWESt				IMPROVe							
	Men				Men				Women			
	*N* = 191				*N* = 639				*N* = 921			
Valence (Sadness/Happiness)	Arousal (Tenseness/Calmness)				Arousal (Tenseness/Calmness)				Arousal (Tenseness/Calmness)			
	Calmer	No change	Tenser	**Total**	Calmer	No change	Tenser	**Total**	Calmer	No Change	Tenser	**Total**
Happier	19	13	11	**43**	43	37	28	**108**	55	35	28	**118**
No Change	20	53	19	**92**	52	215	65	**332**	58	260	100	**418**
Sadder	7	12	37	**56**	33	56	110	**199**	46	97	242	**385**
**Total**	**46**	**78**	**67**	**191**	**128**	**308**	**203**	**639**	**159**	**392**	**370**	**921**

*GWESt* Gulf War I Era study, *IMPROVe* Interviews to Measure PTSD Recovery of Seterans study

**Bold face font** represents row and column totals

Results Reported as Ns

## Discussion

The probability of undesirable affective change in the present study ranged from 29.3 to 41.8%. This proportion was near the upper range of individuals reporting unexpected upset in a systematic review [9], and could reflect the fact that our samples were drawn from individuals highly impaired by PTSD. Strong, negative emotional responses to trauma reminders are a core feature of PTSD. These samples may therefore represent the upper bounds for risks of emotional upset to mailed, trauma-related research. Even so, most respondents had no change in their affect post-survey, and approximately one eighth to one fifth of participants actually reported being happier or calmer. Changes in valence and arousal did not necessarily move in lock-step, so that some individuals became both happier and tenser post-survey, while others became sadder yet calmer. Given the notable minority of participants who experienced an undesirable affective change, it is particularly important to note that our data suggest that the magnitude of upset experienced was small. Even in the subgroups with highest risk of undesirable affective change, such as women with a military sexual assault history, mean score changes tended to be less than one “half-manikin.” Participants’ net affective change in the present study was approximately one tenth to one half that reported by college students when they looked at magazine advertisements with or without background music [26]. Likewise, effect sizes as measured by Cohen’s *d* tended to fall in the range considered small [25] and were similar to that reported by university students before and after reading a pamphlet about using nonsexist language in educational publications [27]. By contrast, walking outdoors for 10 min has been associated with medium to large effects sizes on the SAM (Cohen’s *d* = 0.46 for arousal and 1.12 for valence) [27].

Pre- and post-test evaluations of participants’ reactions to trauma-related surveys are regrettably rare—we know of only three others—but their findings are similar to our own. After completing an in-clinic, self-administered questionnaire about combat exposures and PTSD symptoms, 76 Veterans with PTSD had a 0.50 net increase in their sadness and a 0.26 net increase in their tenseness using the SAM—again, less than one “half-manikin” [28]. Two studies of college undergraduates showed that survey participants had only negligible changes in anxiety, anger, and depression after completing in-class questionnaires about sexual abuse and behaviors [29, 30]. Whether any of these studies can be generalized to mailed surveys, where individuals typically do not agree to receive a questionnaire in advance, is uncertain.

Also uncertain is the risk associated with receiving surveys without trauma-related content. To meet IRBs’ “minimal risk” criteria, the probability and magnitude of harm associated with the research cannot exceed that of day-to-day living or physical or psychological examinations or tests. But what is the risk and magnitude of emotional upset to receiving a marketing survey in the mail? Or a survey from a political party to which one does not belong? What are the risks and magnitudes of emotional upset to physical and psychological examinations? To our knowledge, none of these routine, day-to-day risks have been quantified. In their absence, it is difficult to place results emerging from trauma-related fields into context. We know of only one study to examine participants’ emotional reactions to a survey without trauma content, for example [28]. In that study, 62 Veterans with PTSD completed an in-clinic questionnaire about their personality, personal values, and satisfaction with their health care. According to the first author, almost 12% reported being unexpectedly upset by the survey (A. Ferrier-Auerbach, electronic communication, Aug. 29, 2016). Although some Veterans were upset after the survey, participants as a whole had a 0.08-point net reduction in their sadness post-survey and a 0.41-point net reduction in their tenseness as measured by the SAM. This small sized study suggests that survey research unrelated to trauma is not necessarily risk-free; however, there did appear to be slight differences in how the participants reacted to surveys with and without trauma content.

In the present study, women with a history of military sexual assault had the largest net change in sadness and tenseness post-survey, although—again—that change represented less than one “half-manikin.” Interestingly, respondents’ reactions could not have been triggered by sexual assault survey items because we did not ask any on the Time 3 questionnaire. As with Jaffe et al.’s [10] meta-analysis, positive PTSD screens had the most consistent effect on probability of undesirable affective change, but the magnitude of change was small. Against expectations, combat history’s association with undesirable affective change was inconsistent across the groups. Persistent serious mental illness had no association with affective change post-survey.

### Strengths and limitations

Our use of a pre- and post-test design allowed us to control for participants’ baseline affect. Findings showed that our hypothesized correlates and predictors of undesirable affect were actually associated with greater baseline sadness and tenseness. Without this baseline, we might have overestimated the impact of combat, sexual assault, PTSD and serious mental illness on participants’ post-survey reactions. We urge caution when interpreting data from single post-test studies. Although each participant served as his or her own control, we did not include an experimental control group, e.g., a no-survey control. Therefore, we cannot say whether the survey or some other factor caused some participants’ affect to change. Some participants may have taken breaks between answering questions on the survey, which would have prolonged the time between the pre- and post-test SAMs and thus potentially introduced maturation effects*.*

“Re-test” effects occur when groups repeatedly responding to the same scale report inexplicable net improvements despite no interventions being offered [31]. It seems unlikely that the present study was affected by this. Participants’ affective change post-survey was heterogeneous and on net opposite the direction expected for re-test effect. Furthermore, substantial numbers of test-takers showed opposing changes on the two SAM scales. Were a re-test phenomenon operating, the direction of change should have been desirable and uniform across test-takers and across the two SAM scales. Random error, which undoubtedly affected the present study, typically does not bias the direction or magnitude of an effect size but does increase imprecision and Type II error. To the extent that some other, unknowable systematic measurement error affected the study, the effect sizes reported here could be under or over estimates of the true effect.

Including 2 samples, which were comprised of 3 unique groups, increased our understanding of the findings’ generalizability. However, no group could be considered representative of the United States’ general population or even of the US Veteran population. Each group had exceptionally high trauma histories. They also had PTSD symptoms sufficiently severe to prompt them to seek disability benefits. These characteristics might have caused them to react more negatively to a trauma-related survey than individuals without PTSD or with less disabling PTSD symptoms.

Labott et al. [32] has found that most participants upset by disclosing traumatic experiences in telephone interviews require 0–0.5 h to “get over” their upset, while a few respondents require as much as 72 h. This study assessed participants’ change in emotion immediately after completing a trauma-related survey, but we did not assess the duration of that change. Future studies should consider both the magnitude and duration of undesirable affective change.

The net change in negative affect in the present study was less than that reported for many activities of daily living, suggesting that expedited IRB procedures may be appropriate for trauma-related surveys. This is not the same as saying that trauma-related surveys are risk-free, and it should not be taken to mean that researchers need not concern themselves with an appropriate safety plan for research participants. Our own safety plan explicitly encouraged Veterans to opt out of the study if they felt it might be too upsetting. Therefore, we may have selected out those Veterans at highest risk for an extreme reaction to the questionnaires.

Compared to other measures of affect, such as Emocards [33] or smartphone applications, research participants find the SAM relatively easy to use and interpret [34, 35]. Unfortunately, despite the SAM’s widespread use for assessing emotional responses to stimuli, state norms are not readily available. Our participants’ baseline valence was similar to that reported for Veterans being seen in a PTSD clinic [28] and approximately 2 full points (or one “full manikin”) sadder than that reported for 92 Spanish University students [35]. Our participants’ baseline arousal was slightly tenser than the clinic Veterans’ baseline, but similar to the University students’ baseline [28, 35]. Clinically meaningful changes on the SAM have not been defined—a limitation that unfortunately applies to most state measures of affect. Without such definitions, one must rely on less satisfying estimates of effect size, such as Cohen’s *d*, or on one’s own personal belief as to whether a “half-manikin” or “full-manikin” change is meaningful.

## Conclusions

Although a substantial minority of Veterans in these studies reported more sadness or tenseness after completing a trauma-related survey, the net change in undesirable affect was similar to that reported for individuals before and after reading a grammar pamphlet and less than the affective change associated with taking a walk or looking at magazine ads with and without background music. Some respondents became happier or calmer post-survey. Most hypothesized predictors and correlates of undesirable affective change were actually associated with higher baseline scores on the SAM and did not necessarily translate into larger, undesirable changes post-survey. Our data do not suggest that separate, special protections need to be created for Veterans with PTSD, persistent serious mental illness, combat exposure, or military sexual assault history when receiving unsolicited, trauma-related surveys by mail. Because our study samples were arguably at greater risk for emotional upset than more general populations, these results are cautiously reassuring. Research into the risks and harms of mailed, trauma-related survey research could be considerably strengthened by using more pre- and post- test designs with experimental control groups, by clarifying the risks and harms of mailed surveys that do not ask about trauma, and by defining meaningful clinical changes in outcome measures.

## Additional files

Additional file 1: Supplementary material: eFigure. “Mean Change in Affect by Affective Score Change Category.” (DOCX 105 kb)

Additional file 2: Supplementary material: eTable. “Baseline (Pre-Survey) and Post-Survey Valence and Arousal Scores by Hypothesized Correlates or Predictors. Results Reported as Means and Standard Deviations (SD).” (DOCX 21 kb)

## Abbreviations

GWESt: Gulf War 1 Era Veterans study
IMPROVe: Interviews to Measure PTSD Recovery of Veterans study
IRB: Institutional review board
PTSD: Posttraumatic stress disorder
RRPQ: Reactions to Research Participation Questionnaire
SAM: Self-Assessment Manikin
SD: Standard deviations
VA: United States Department of Veterans Affairs
