# Supplementary material for: Changes in affect after completing a mailed survey about trauma: two pre- and post-test studies in former disability applicants for posttraumatic stress disorder
Source: BMC Med Res Methodol. 2017 May 10;17:81. doi: 10.1186/s12874-017-0357-x (PMC5424391; doi:10.1186/s12874-017-0357-x)
Supplement: Supplementary file 1 — Supplementary material: eFigure. “Mean Change in Affect by Affective Score Change Category.” (DOCX 105 kb) [file 12874_2017_357_MOESM1_ESM.docx]

|  | **GWEST Men** | **IMPROVe Men** | **IMPROVe Women** |  |
| --- | --- | --- | --- | --- |
| Valence  Mean Score Change (SD) | 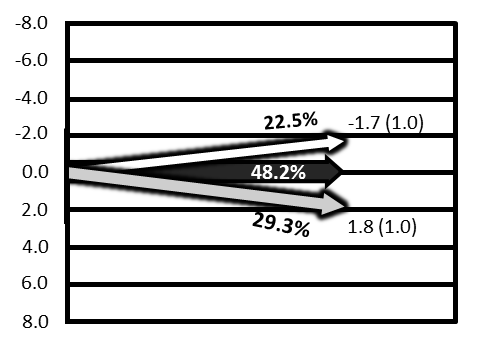 | 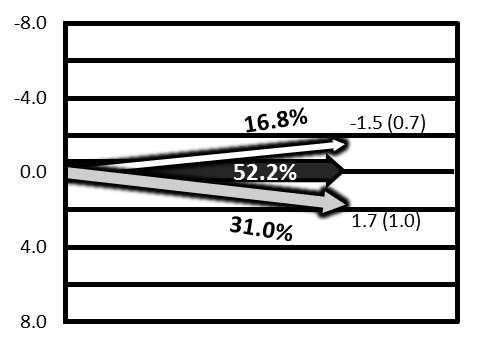 | 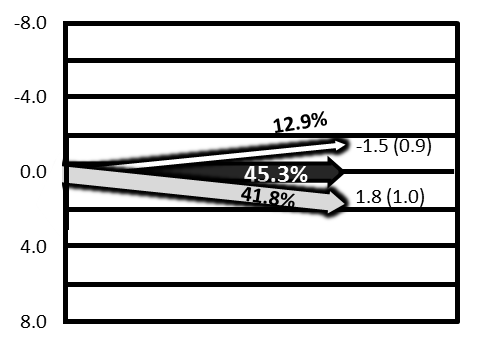 | Happier  Sadder |
|  |  |  |  |  |
| Arousal  Mean Score Change (SD) | 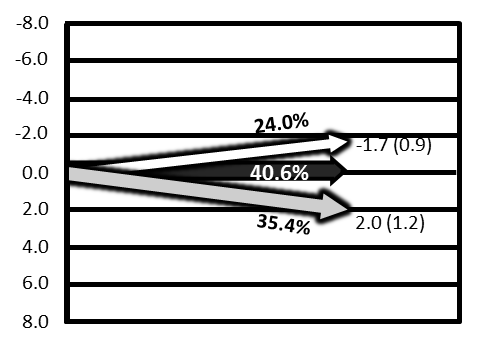 | 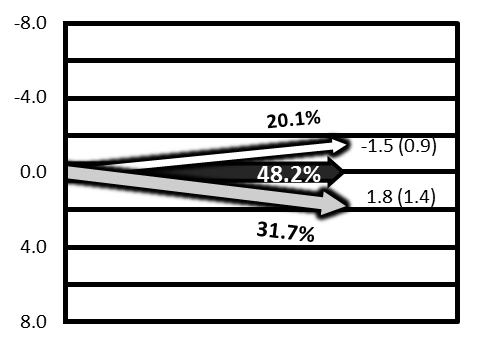 | 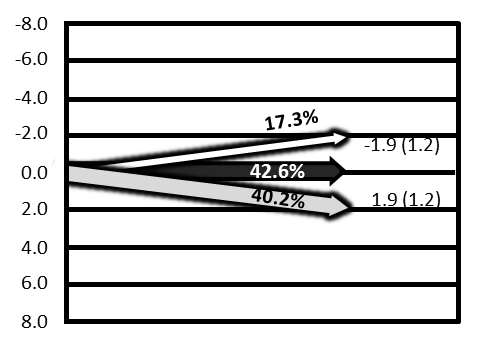 | Calmer  Tenser |
|  |  |  | 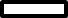 Happier or Calmer  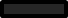 No Change  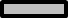 Sadder or Tenser |  |
