# Supplementary material for: Changes in affect after completing a mailed survey about trauma: two pre- and post-test studies in former disability applicants for posttraumatic stress disorder
Source: BMC Med Res Methodol. 2017 May 10;17:81. doi: 10.1186/s12874-017-0357-x (PMC5424391; doi:10.1186/s12874-017-0357-x)
Supplement: Supplementary file 2 — Supplementary material: eTable. “Baseline (Pre-Survey) and Post-Survey Valence and Arousal Scores by Hypothesized Correlates or Predictors. Results Reported as Means and Standard Deviations (SD).” (DOCX 21 kb) [file 12874_2017_357_MOESM2_ESM.docx]

eTable. Baseline (Pre-Survey) and Post-Survey Valence and Arousal Scores by Hypothesized Correlates or Predictors. Results Reported as Means and Standard Deviations (SD)

| Correlates and Predictors | Self-Assessment Manikin Item | | | | | | | |
| --- | --- | --- | --- | --- | --- | --- | --- | --- |
|  | Valence  (Sadness/Happiness) | | | | Arousal  (Tenseness/Calmness) | | | |
|  | Baseline (Pre-Survey) | | Post-Survey | | Baseline (Pre-Survey) | | Post-Survey | |
|  | With Attribute | Without Attribute | With Attribute | Without Attribute | With Attribute | Without Attribute | With Attribute | Without Attribute |
| PTSD screen positive |  |  |  |  |  |  |  |  |
| GWESt Men | 6.4 (1.5) | 4.3 (1.8)*** | 6.2 (1.8) | 4.0 (1.9)*** | 5.9 (1.8) | 4.1 (1.8)*** | 5.9 (1.9) | 4.0 (2.0)*** |
| IMPROVe Men | 6.0 (1.6) | 4.3 (1.8)*** | 6.3 (1.5) | 4.4 (1.7)*** | 5.7 (1.7) | 4.1 (1.9)*** | 6.0 (1.8) | 4.2 (1.8)*** |
| IMPROVe Women | 5.8 (1.7) | 3.9 (1.7)*** | 6.4 (1.5) | 4.2 (1.7)*** | 5.7 (1.8) | 3.9 (1.8)*** | 6.2 (1.8) | 4.2 (1.8)*** |
| Persistent SMI |  |  |  |  |  |  |  |  |
| IMPROVe Men | 5.9 (1.6) | 5.4 (1.8) | 6.2 (1.7) | 5.7 (1.8)* | 5.8 (1.9) | 5.1 (1.9)** | 6.1 (1.7) | 5.4 (1.9)** |
| IMPROVe Women | 5.3 (1.9) | 4.9 (1.9)* | 5.8 (2.0) | 5.5 (1.9)* | 5.3 (2.2) | 4.9 (1.9)** | 5.7 (2.1) | 5.4 (2.0)* |
| Combat exposure |  |  |  |  |  |  |  |  |
| GWESt Men | 5.7 (1.8) | 5.4 (1.9) | 6.1 (1.8) | 5.3 (2.1)** | 5.3 (2.0) | 5.1 (2.4) | 5.8 (2.0) | 5.3 (2.2) |
| IMPROVe Men | 5.5 (1.8) | 4.9 (1.8)* | 5.8 (1.8) | 5.0 (1.6)** | 5.3 (1.9) | 4.4 (1.7)** | 5.5 (2.0) | 4.7 (1.8)** |
| IMPROVe Women | 5.2 (2.0) | 5.0 (1.9) | 5.6 (1.9) | 5.5 (1.9) | 5.2 (2.0) | 4.9 (2.0)* | 5.4 (2.0) | 5.4 (2.0) |
| Military sexual assault history |  |  |  |  |  |  |  |  |
| GWESt Men | 6.7 (1.7) | 5.6 (1.9) | 6.1 (2.6) | 5.7 (2.0) | 6.7 (1.5) | 5.2 (2.0)* | 6.1 (2.8) | 5.5 (21) |
| IMPROVe Men | 5.6 (1.7) | 5.5 (1.8) | 5.8 (1.7) | 5.7 (1.8) | 5.2 (1.8) | 5.2 (1.9) | 5.5 (2.0) | 5.5 (2.0) |
| IMPROVe Women | 5.0 (1.9) | 4.9 (2.0) | 5.7 (1.9) | 5.2 (1.9)** | 5.0 (2.0) | 4.9 (2.0) | 5.6 (2.0) | 5.0 (2.0)*** |

GWESt=Gulf War I Era Veterans Study. IMPROVe = Interviews to Measure PTSD Recovery of Veterans Study. PTSD = Posttraumatic stress disorder. SMI = Serious mental illness. Statistical tests compare those with and without the attribute. *p < 0.05, **p < 0.01, ***p < 0.001
